# Supplementary material for: Optimization of Extraction Conditions to Improve Phenolic Content and In Vitro Antioxidant Activity in Craft Brewers’ Spent Grain Using Response Surface Methodology (RSM)
Source: Foods. 2020 Oct 2;9(10):1398. doi: 10.3390/foods9101398 (PMC7599942; doi:10.3390/foods9101398)
Supplement: Supplementary file 1 [file foods-09-01398-s001.pdf]

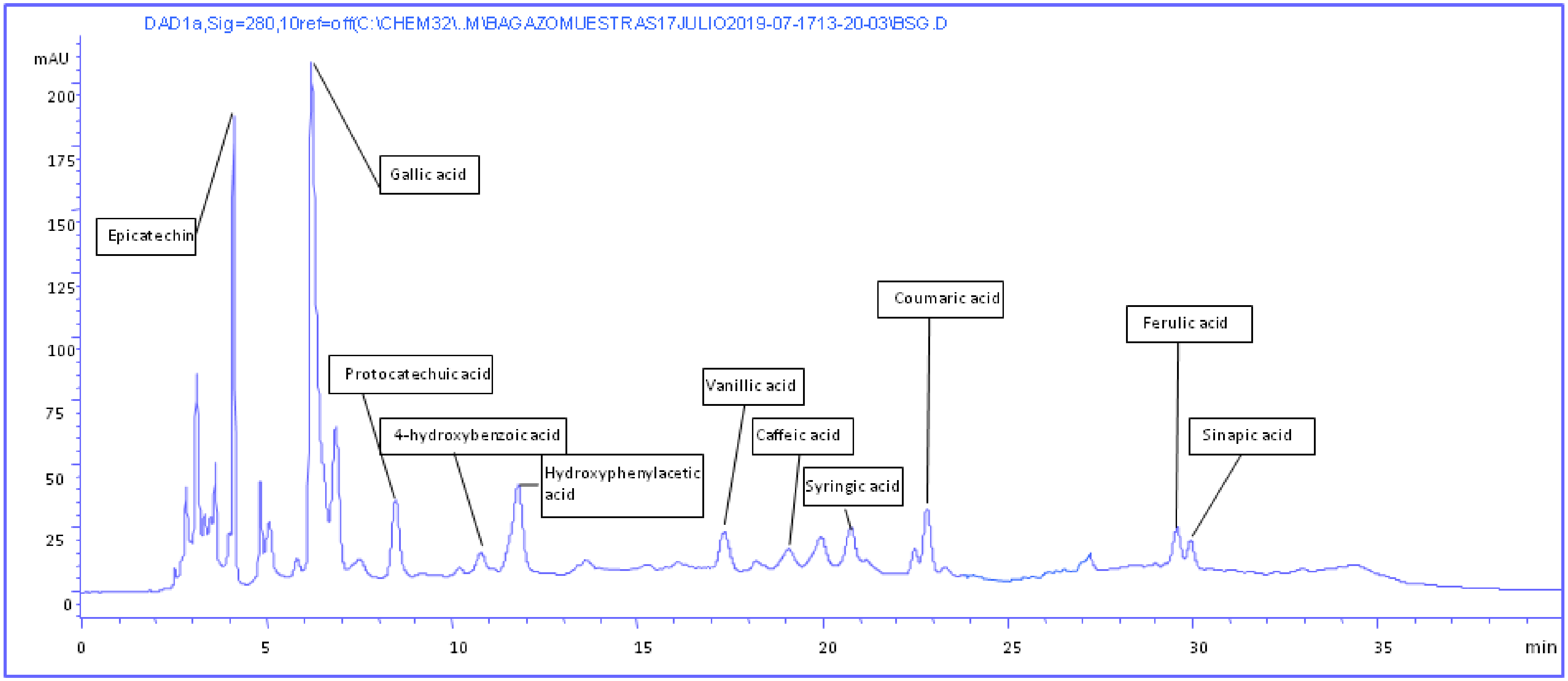


**Figure S1.** HPLC chromatogram of BSG extract.

**Table S1.** Standard calibration curves for Epicatechin in BSG samples.

| Standard | Concentration (µg/ml] | Area (mAU) | Regression formula | $R^2$ |
|---|---|---|---|---|
| Epicatechin | 100 | 51236,6 | y=543,08x | 0,9864 |
| Epicatechin | 75 | 44280,6 | | |
| Epicatechin | 50 | 27737,2 | | |
| Epicatechin | 25 | 13873 | | |
| Epicatechin | 10 | 5872,6 | | |

**Table S2.** Standard calibration curves for Gallic acid in BSG samples.

| Standard | Concentration (µg/ml] | Area (mAU) | Regression formula | $R^2$ |
|---|---|---|---|---|
| Gallic acid | 100 | 6697,1 | y = 67,196x | 0,9989 |
| Gallic acid | 75 | 5115,4 | | |
| Gallic acid | 50 | 3367,6 | | |
| Gallic acid | 25 | 1558,1 | | |
| Gallic acid | 10 | 595,6 | | |

**Table S3.** Standard calibration curves for Procatechuic acid in BSG samples.

| Standard | Concentration (µg/ml] | Area (mAU) | Regression formula | $R^2$ |
|---|---|---|---|---|
| Procatechuic acid | 100 | 4521 | y = 46,134x | 0,9981 |
| Procatechuic acid | 75 | 3507,6 | | |
| Procatechuic acid | 50 | 2391 | | |
| Procatechuic acid | 25 | 1209 | | |
| Procatechuic acid | 10 | 467,2 | | |

**Table S4.** Standard calibration curves for Hydroxybenzoic acid in BSG samples.

| Standard | Concentration (µg/ml] | Area (mAU) | Regression formula | $R^2$ |
|---|---|---|---|---|
| Hydroxybenzoic acid | 100 | 4454 | y = 44,822x | 0,9998 |
| Hydroxybenzoic acid | 75 | 3389,1 | | |
| Hydroxybenzoic acid | 50 | 2261,9 | | |
| Hydroxybenzoic acid | 25 | 1107,4 | | |
| Hydroxybenzoic acid | 10 | 453,5 | | |

**Table S5.** Standard calibration curves for Hydroxyphenylacetic acid in BSG samples.

| Standard | Concentration (µg/ml] | Area (mAU) | Regression formula | $R^2$ |
|---|---|---|---|---|
| Hydroxyphenylacetic acid | 100 | 1196,8 | y = 11,92x | 0,9994 |
| Hydroxyphenylacetic acid | 75 | 901,5 | | |
| Hydroxyphenylacetic acid | 50 | 584,4 | | |
| Hydroxyphenylacetic acid | 25 | 283,1 | | |
| Hydroxyphenylacetic acid | 10 | 111 | | |

**Table S6.** Standard calibration curves for Vanillic acid in BSG samples.

| Standard | Concentration (µg/ml] | Area (mAU) | Regression formula | $R^2$ |
|---|---|---|---|---|
| Vanillic acid | 100 | 4993,1 | y = 50,18x | 0,9994 |
| Vanillic acid | 75 | 3828,3 | | |
| Vanillic acid | 50 | 2491,2 | | |
| Vanillic acid | 25 | 1209,3 | | |
| Vanillic acid | 10 | 465,9 | | |

**Table S7.** Standard calibration curves for Caffeic acid in BSG samples.

| Standard | Concentration (µg/ml] | Area (mAU) | Regression formula | $R^2$ |
|---|---|---|---|---|
| Caffeic acid | 100 | 8269,38 | y = 83,343x | 0,9997 |
| Caffeic acid | 75 | 6312 | | |
| Caffeic acid | 50 | 4218,8 | | |
| Caffeic acid | 25 | 2065,1 | | |
| Caffeic acid | 10 | 811,581 | | |

**Table S8.** Standard calibration curves for Syringic acid in BSG samples.

| Standard | Concentration (µg/ml] | Area (mAU) | Regression formula | $R^2$ |
|---|---|---|---|---|
| Syringic acid | 100 | 8462,3 | y = 85,064x | 0,9999 |
| Syringic acid | 75 | 6423,7 | | |
| Syringic acid | 50 | 4274,4 | | |
| Syringic acid | 25 | 2140,8 | | |
| Syringic acid | 10 | 820,4 | | |

**Table S9.** Standard calibration curves for Coumaric acid in BSG samples.

| Standard | Concentration (µg/ml] | Area (mAU) | Regression formula | $R^2$ |
|---|---|---|---|---|
| Coumaric acid | 100 | 13115,6 | y = 134,81x | 0,9965 |
| Coumaric acid | 75 | 10531,7 | | |
| Coumaric acid | 50 | 6765,5 | | |
| Coumaric acid | 25 | 3507,1 | | |
| Coumaric acid | 10 | 1382,27 | | |

**Table S10.** Standard calibration curves for Transferulic acid in BSG samples.

| Standard | Concentration (µg/ml] | Area (mAU) | Regression formula | $R^2$ |
|---|---|---|---|---|
| Transferulic acid | 100 | 7692,63 | y = 77,156x | 0,9995 |
| Transferulic acid | 75 | 5844,34 | | |
| Transferulic acid | 50 | 3871,05 | | |
| Transferulic acid | 25 | 1849,61 | | |
| Transferulic acid | 10 | 701,7 | | |

**Table S11.** Standard calibration curves for Sinapic acid in BSG samples.

| Standard | Concentration (µg/ml] | Area (mAU) | Regression formula | $R^2$ |
|---|---|---|---|---|
| Sinapic acid | 100 | 3531,2 | y = 35,881x | 0,9984 |
| Sinapic acid | 75 | 2723,15 | | |
| Sinapic acid | 50 | 1870,4 | | |
| Sinapic acid | 25 | 886,1 | | |
| Sinapic acid | 10 | 333,4 | | |
